# Supplementary material for: Resurgence of Omicron BA.2 in SARS-CoV-2 infection-naive Hong Kong
Source: Nat Commun. 2023 Apr 27;14:2422. doi: 10.1038/s41467-023-38201-5 (PMC10134727; doi:10.1038/s41467-023-38201-5)
Supplement: Supplementary file 1 — Supplementary Information [file 41467_2023_38201_MOESM1_ESM.pdf]

# Supplementary Information

# Resurgence of Omicron BA.2 in SARS-CoV-2 infection-naive Hong Kong

Ruopeng Xie[1,2,6], Kimberly M. Edwards[1,2,6], Dillon C. Adam[1], Kathy S.M. Leung[1], Tim K. Tsang[1], Shreya Gurung[1,2], Weijia Xiong[1], Xiaoman Wei[1,2], Daisy Y.M. Ng[1], Gigi Y.Z. Liu[1], Pavithra Krishnan[1], Lydia D.J. Chang[1], Samuel M.S. Cheng[1], Haogao Gu[1], Gilman K.H. Siu[3], Joseph T. Wu[1,4], Gabriel M. Leung[1,4], Malik Peiris[1,5], Benjamin J. Cowling[1,4], Leo L.M. Poon[1,2,5], Vijaykrishna Dhanasekaran[1,2,*]

**Affiliations:**

[1]School of Public Health, LKS Faculty of Medicine, The University of Hong Kong; Hong Kong S.A.R., China.

[2]HKU-Pasteur Research Pole, School of Public Health, LKS Faculty of Medicine, The University of Hong Kong; Hong Kong S.A.R., China

[3]Department of Health Technology and Informatics, The Hong Kong Polytechnic University; Hong Kong S.A.R., China.

[4]Laboratory of Data Discovery for Health, Hong Kong Science and Technology Park, New Territories, Hong Kong S.A.R., China

[5]Centre for Immunology & Infection, Hong Kong Science and Technology Park, New Territories, Hong Kong S.A.R., China.

[$]These authors contributed equally: Ruopeng Xie, Kimberly M. Edwards.

[*]Corresponding author. Email: veej@hku.hk

Supplementary Notes:

## Sensitivity analysis of $R_e$ estimation

The number of cases sequenced from January to April in Hong Kong was disproportionate (Supplementary Table 1), and as $R_e$ estimation is sensitive to bias from sampling proportions, we conducted sensitivity tests on the sampling proportion prior choice in the BDSS model by giving a uniform distribution as prior with the upper bounds at 0.001, 0.01, 0.1, 0.5 and 1. As a result, the estimation of sampling proportion converged at 0.0002, 0.008, 0.52, 0.311 and 0.505, corresponding to very different values of $R_e$ during the initial stages of resurgence (10-24 January 2022) (Supplementary Fig. 9). Our sensitivity analysis showed unreliable priors could lead to convergent high sampling proportions, overestimating $R_e$ unexpectedly.

The sampling of sequences in constructing $R_e$ is also important. In this study, we tested the effect of subsampling using two sampling schemes (uniform and proportional), as recommended by the WHO for practical use in different settings and scenarios [1]. We constructed three datasets (n = 262, uniform: 20 sequences per week; n = 502, uniform: 40 sequences per week; n = 897, proportional) (Supplementary Fig. 11 and Supplementary Table 1). Our results showed a good crossover within the confidence intervals and no potential bias in estimations of $R_e$ using different subsampling schemes (Supplementary Fig. 10).

## Assessing the performance of real-time epidemic forecasts based on $R_t$ and $R_e$

Early in outbreaks, it is often difficult to estimate effective reproduction numbers accurately. Herein, we investigated how well $R_e$ and $R_t$ can sequentially reproduce the dynamics of the initial fifth wave of SARS-CoV-2 (10-24 January) in Hong Kong using probabilistic forecasting metrics. Following [2] and [3], the number of new infections at time t could be roughly (superspreading not considered) inferred by multiplying the reproduction number by the total infectiousness of infected individuals at time t, given by the sum of infection incidence up to time step t − 1, weighted by the infectivity function based on the gamma probability distribution ($w_t$) of an individual's infectivity profile once infected [3]. Based on estimated incidence and observed reported cases, the forecasting metrics of absolute error, calibration, and sharpness were calculated implemented in the R package "scoringutils" [4] [5]. Specifically, for $R_e$, we used sequencing time series under various sampling proportion priors to replace the observed reported cases for this projection. As the sampling proportion is over 100% for 17-23 January, we assumed that all reported cases were sequenced. Sampling proportion priors with 0.1, 0.3, 0.5

and 0.7 for 10-16 January were tested. Our results showed large differences between estimated incidence from $R_e$ under low sampling proportion priors (0.1) and sequencing time series (Supplementary Fig. 4). Nevertheless, those projections from $R_t$ and $R_e$ (sampling proportion > 0.3) were able to reproduce this incidence data to some extent, suggesting well-calibrated transmissibility estimations in this study, marked by low absolute error, high calibration, and low sharpness (Supplementary Fig. 4).

## Selection pressure analysis

We investigated the selective forces acting on the spike gene in the BA.2.2 sub lineage sequences in Hong Kong and compared with other BA.2 sub lineages primarily dominated in one specific region. They include Japan (BA.2.3.1), Scotland (BA.2.8), the United States of America (BA.2.12.1) and South Africa (BA.4 and BA.5). After removing duplicates, 215 Hong Kong BA.2.2, 57 Japan BA.2.3.1, 160 Scotland BA.2.8, 218 USA BA.2.12.1, 188 South Africa BA.4 and 55 South Africa BA.5 spike sequences were included in this analysis. For BA.2.12.1 USA, spike sequences were randomly subsampled from major clades. The rate ratio of synonymous to nonsynonymous substitutions ($d_N/d_S$) for each sub lineage were calculated using the SLAC and FEL methods implemented in HyPhy v.2.5.36 [6].

Our results (Supplementary Table 2) indicate HK-BA.2.2 has undergone a similar level of negative selection pressure ($d_N/d_{S}$ = 0.5705 using MEME and 0.6072 using SLAC) as other local lineages (*e.g.,* BA.2.3.1 in Japan, BA.2.8 in Scotland, and BA.2.12.1 in USA). In contrast, BA.4 ($d_N/d_{S}$ = 1.4201 using MEME and 1.3669 using SLAC) and BA.5 ($d_N/d_{S}$ = 1.7192 using MEME and 1.6491 using SLAC) emerged from South Africa [7] with positive selection pressure and circulated globally two months later (Supplementary Table 2).

## Supplementary Figures:

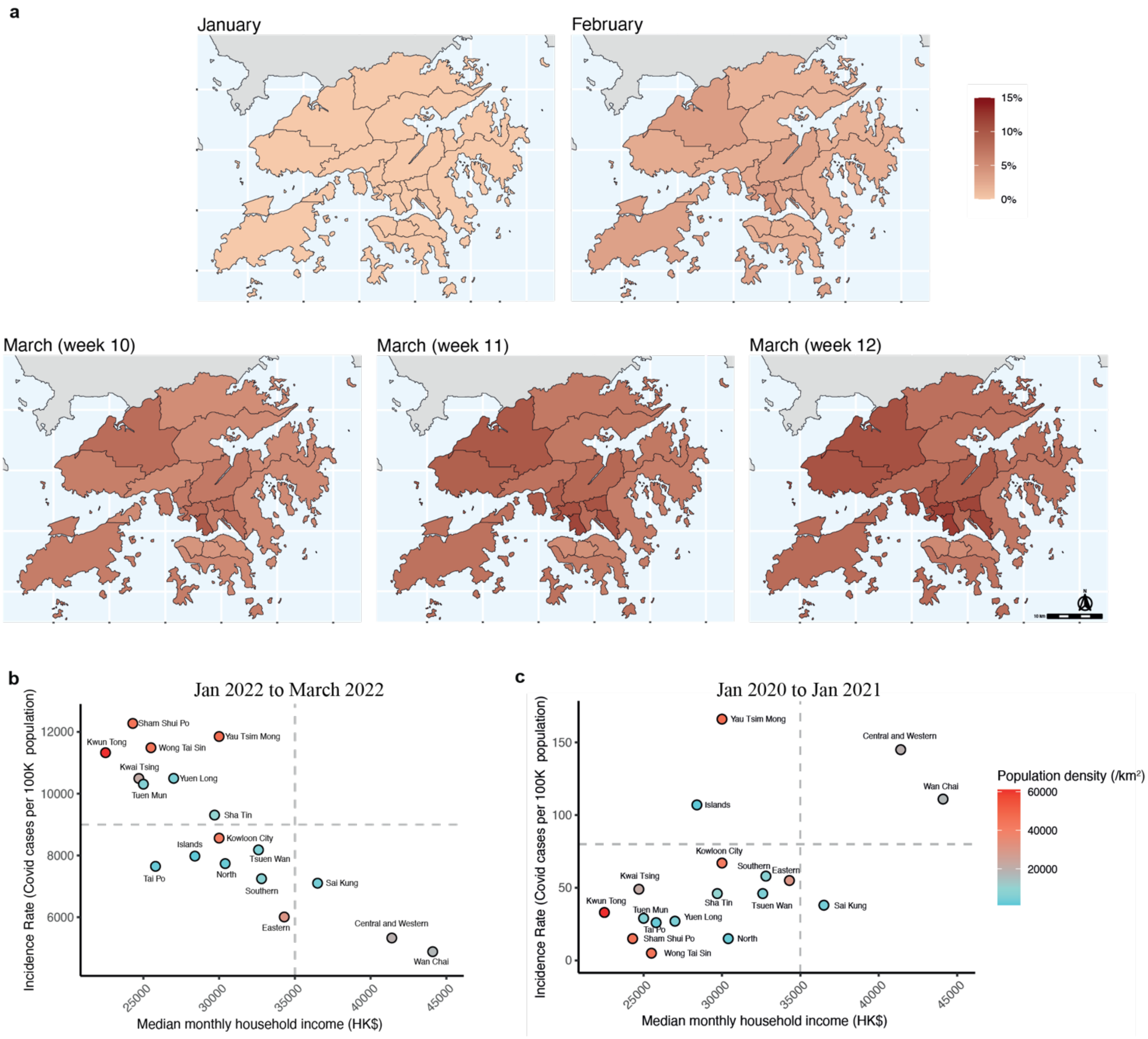


**Supplementary Figure 1** | Geography of SARS-CoV-2 cases in Hong Kong from January to March 2022. (a) Map of Hong Kong's 18 districts shaded by the number of confirmed cases of SARS-CoV-2. Local incidence (b) versus imported incidence (c) by median district household income and colored by population density. Confirmed cases were obtained from Centre for Health Protection (https://www.chp.gov.hk/en/index.html). Maps were generated in R using the package "rnautralearth" (https://CRAN.R-project.org/package=rnaturalearth).

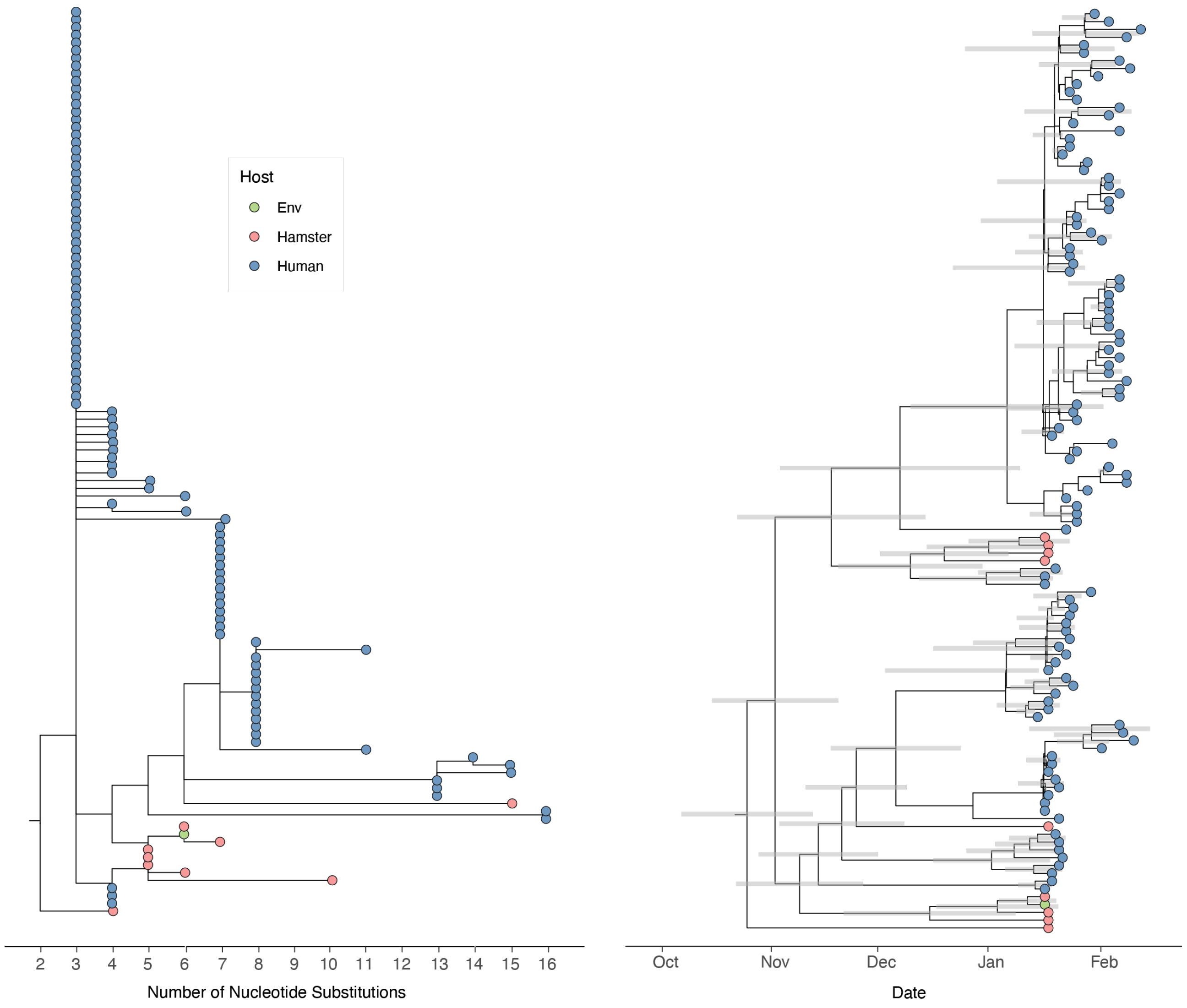


**Supplementary Figure 2** | Evolutionary relationship (maximum likelihood tree on the left and maximum clade credibility tree on right) of HK-AY.127 lineage. Tips colored by hosts. Grey bars denote 95% confidence intervals of estimated time to most recent common ancestor (tMRCA).

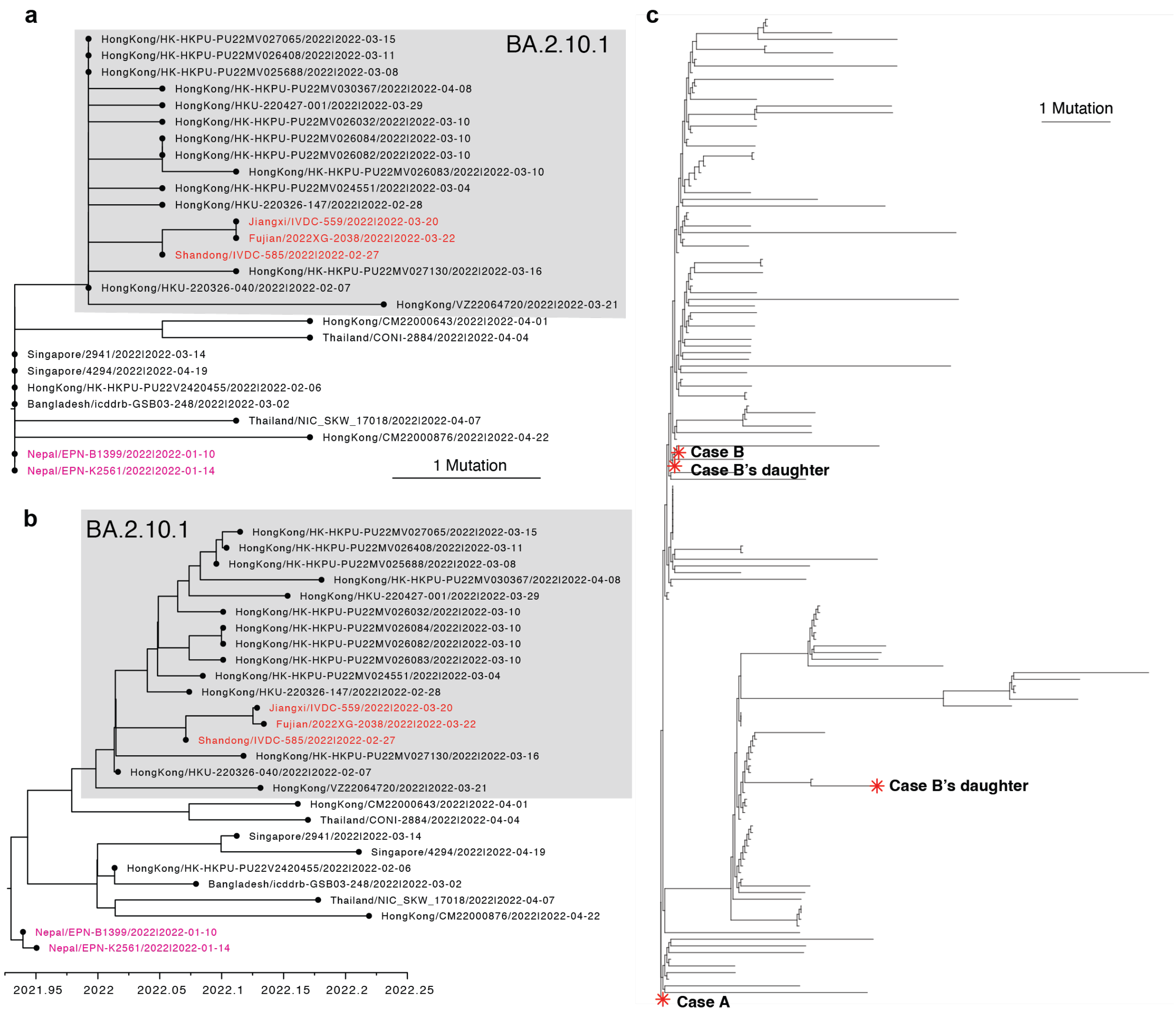


**Supplementary Figure 3** | Evolutionary relationships of BA.2.10.1 and HK-BA.2.2 lineages in Hong Kong. Maximum likelihood tree (a) and dated maximum likelihood tree (b) of BA.2.10.1 after including more sequences from Nepal released on GISAID in June 2022. The grey shaded area represents the BA.2.10.1 monophyletic clade in Hong Kong. Samples collected in mainland China and Nepal are highlighted in red and pink respectively. (c) Maximum likelihood tree of HK-BA.2.2 lineage with the same dataset as **Fig. 1b**. Silka Seaview Hotel cluster is marked with red stars.

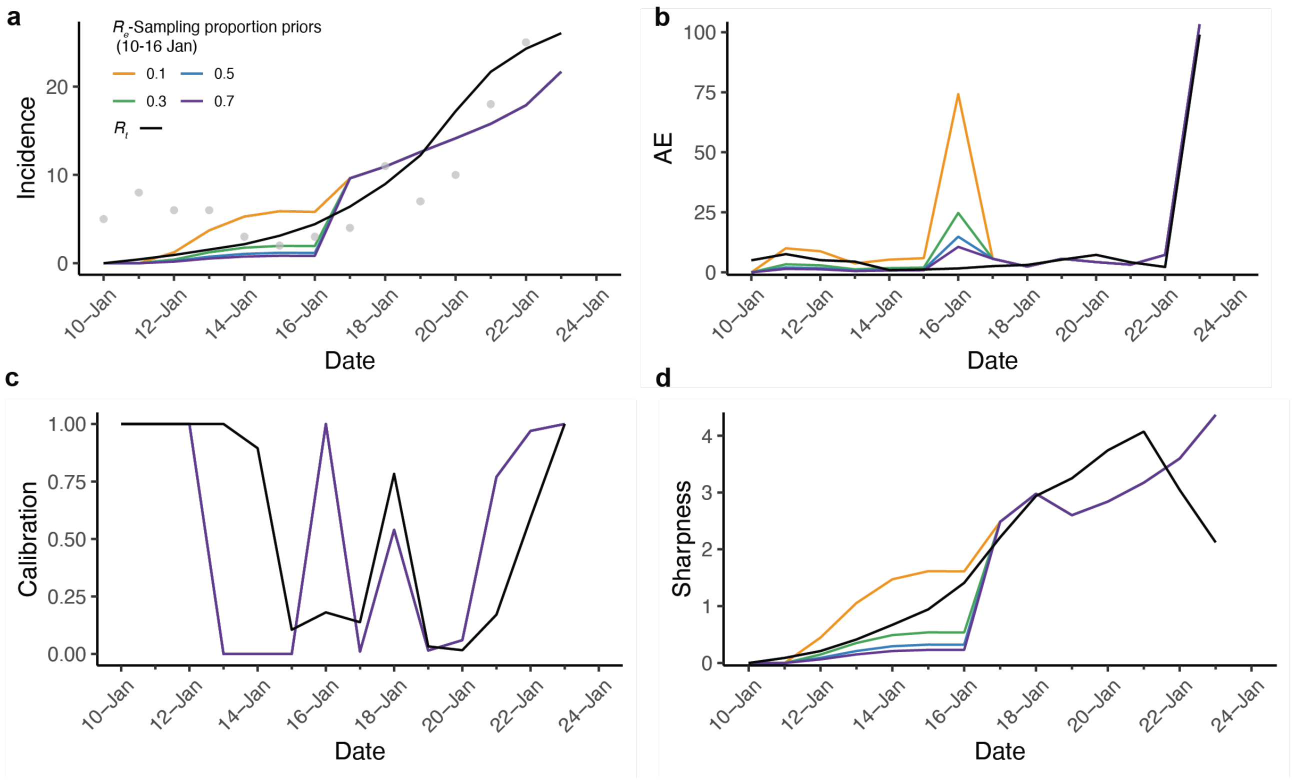


**Supplementary Figure 4** | Forecasting metrics and scores of incidence prediction based on $R_t$ and $R_e$ under various sampling proportion priors. (a) Estimated incidence over time based on $R_t$ (black line) and $R_e$ (colored lines) under various sampling proportion priors. The daily reported number of confirmed cases are shown as grey points. Metrics shown are (b) absolute error (AE, better values closer to 0), (c) calibration (p-value of Anderson-Darling test, greater values indicating better calibration), and (d) sharpness (MAD, sharper models having values closer to 0). When the incidence prediction is > 1 or very different from reported case numbers, the p-value of Anderson-Darling test is equal to 1. Since we only tested different sampling proportion priors for 10-16 January, all lines based on $R_e$ for 17-23 January are coinciding, as well as all lines based on $R_e$ in (c).

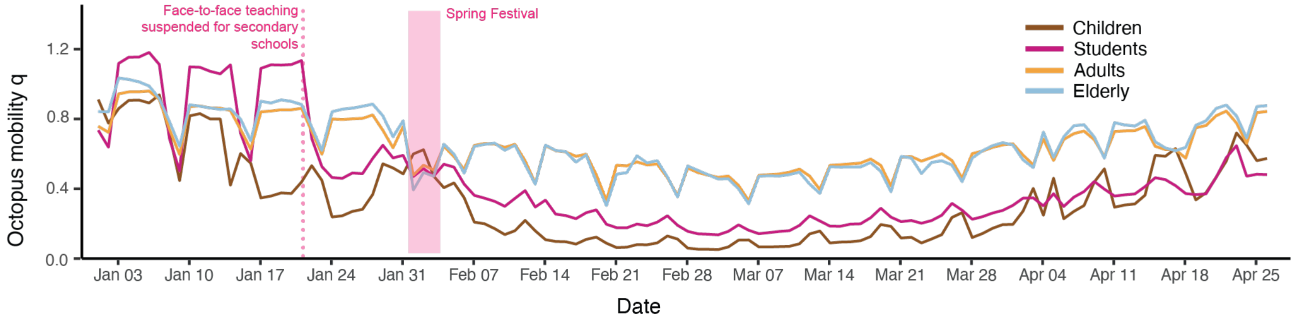


**Supplementary Figure 5** | Octopus (transit card) mobility data in Hong Kong from January 2022 to April 2022.

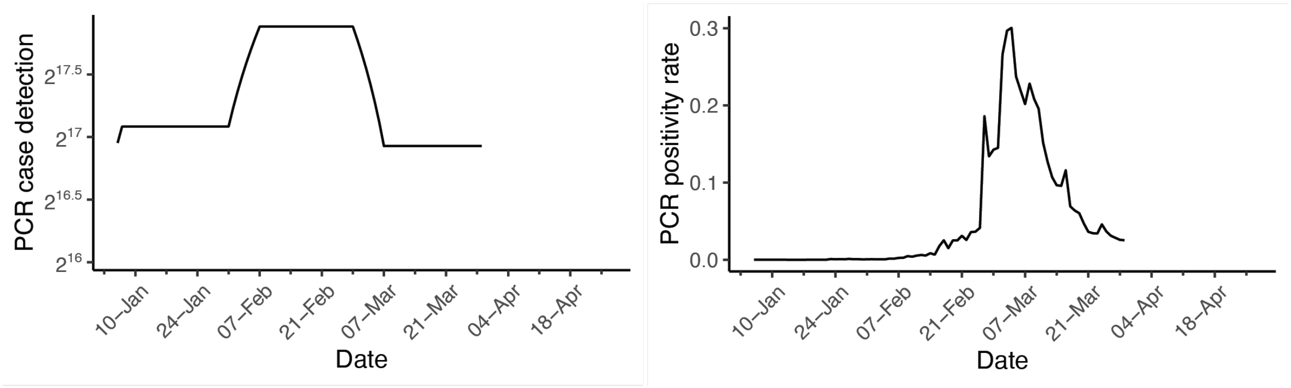


**Supplementary Figure 6** | The number (left) and positivity rate (right) of the PCR tests conducted in Hong Kong from January to March 2022.

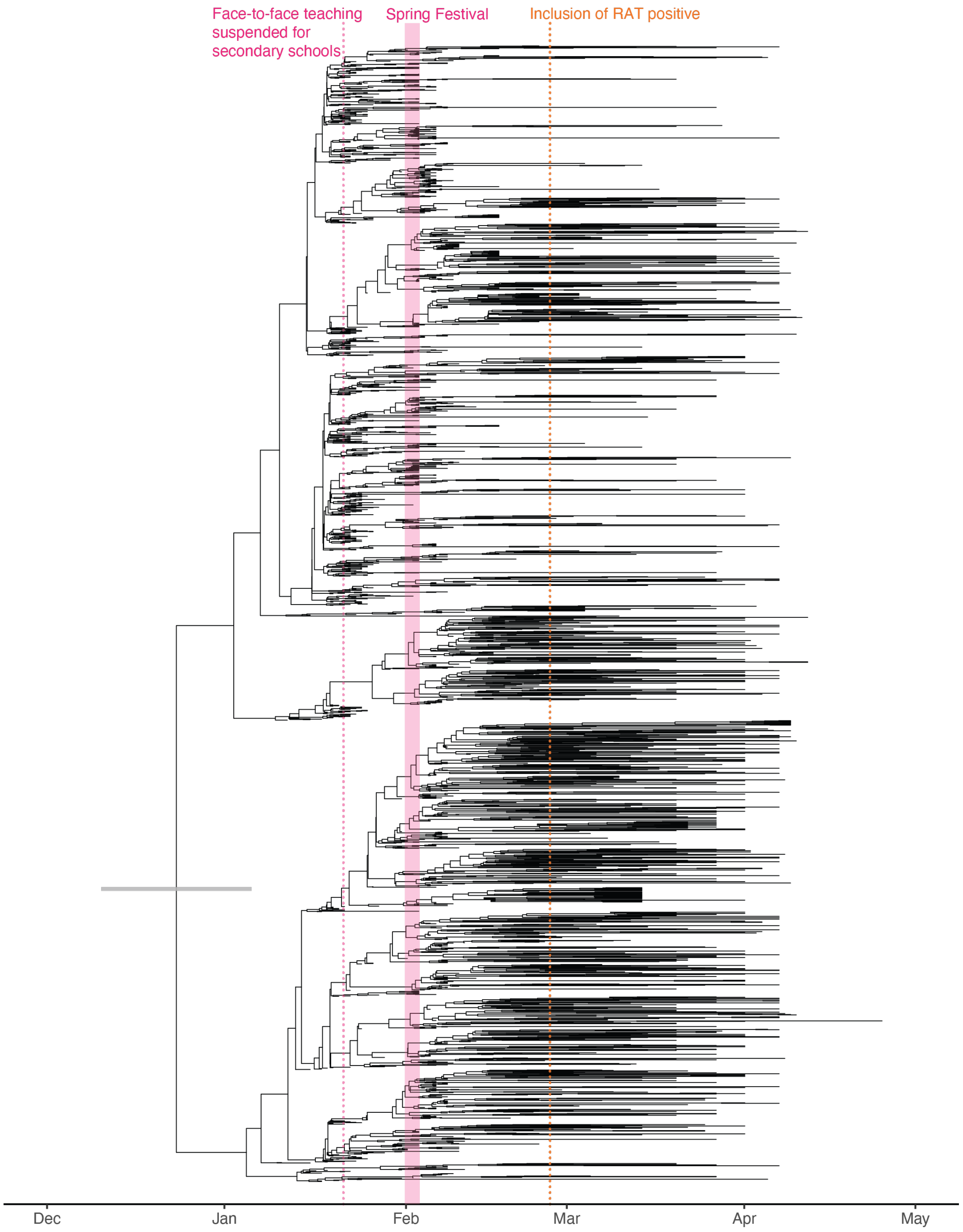


**Supplementary Figure 7** | Time-resolved maximum clade credibility (MCC) tree of BA.2.2 lineage (n = 2,455). The posterior distribution of the root age is shown with the grey bar. The key public health measures and changes in testing strategies are marked in pink and orange.

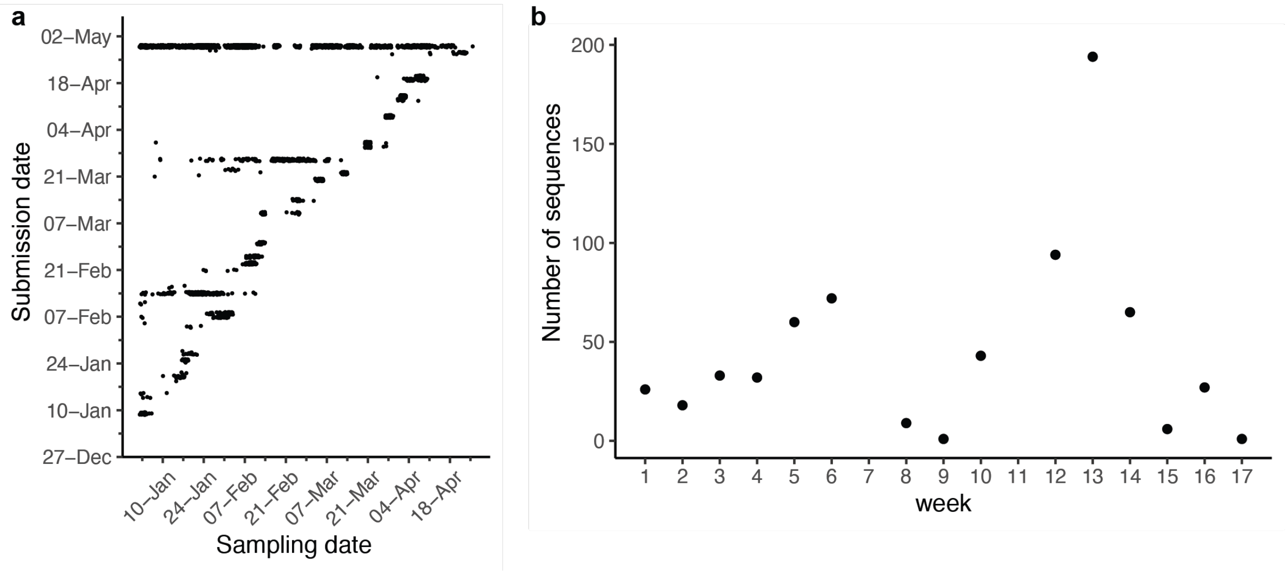


**Supplementary Figure 8** | The collection to submission time lag for SARS-CoV-2 genomes to GISAID from January to April 2022. (a) The sampling date and submission date of sequences. (b) The number of sequences with less than 14 days delay over time. The same epi-week definition was used as in Supplementary fig. 11.

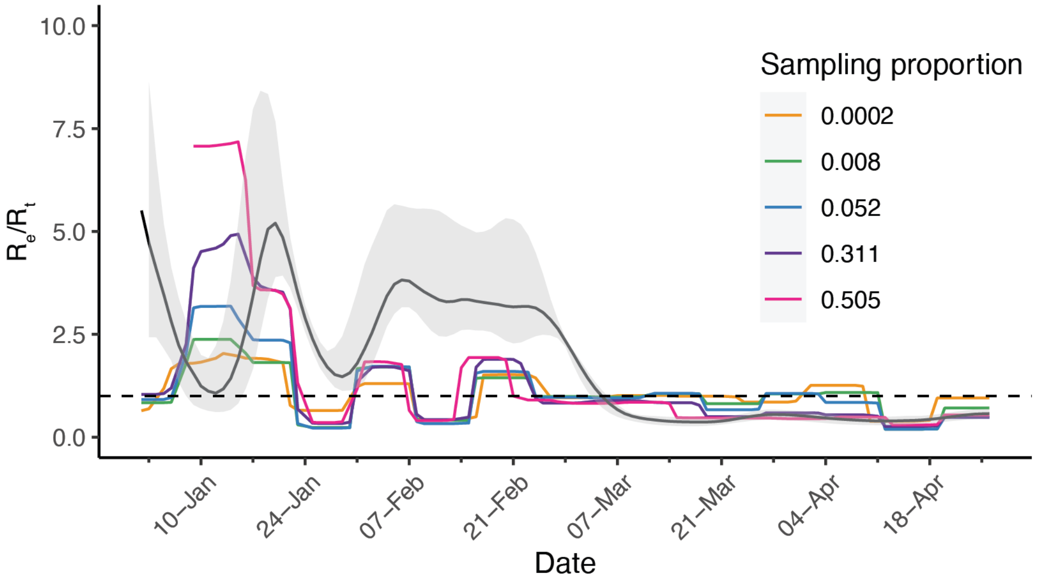


**Supplementary Figure 9** | The effective reproductive number ($R_e$) over time under different sampling proportion assumptions. The instantaneous effective reproduction number ($R_t$) is shown in black line. The shaded area denotes the 95% confidence interval of $R_t$.

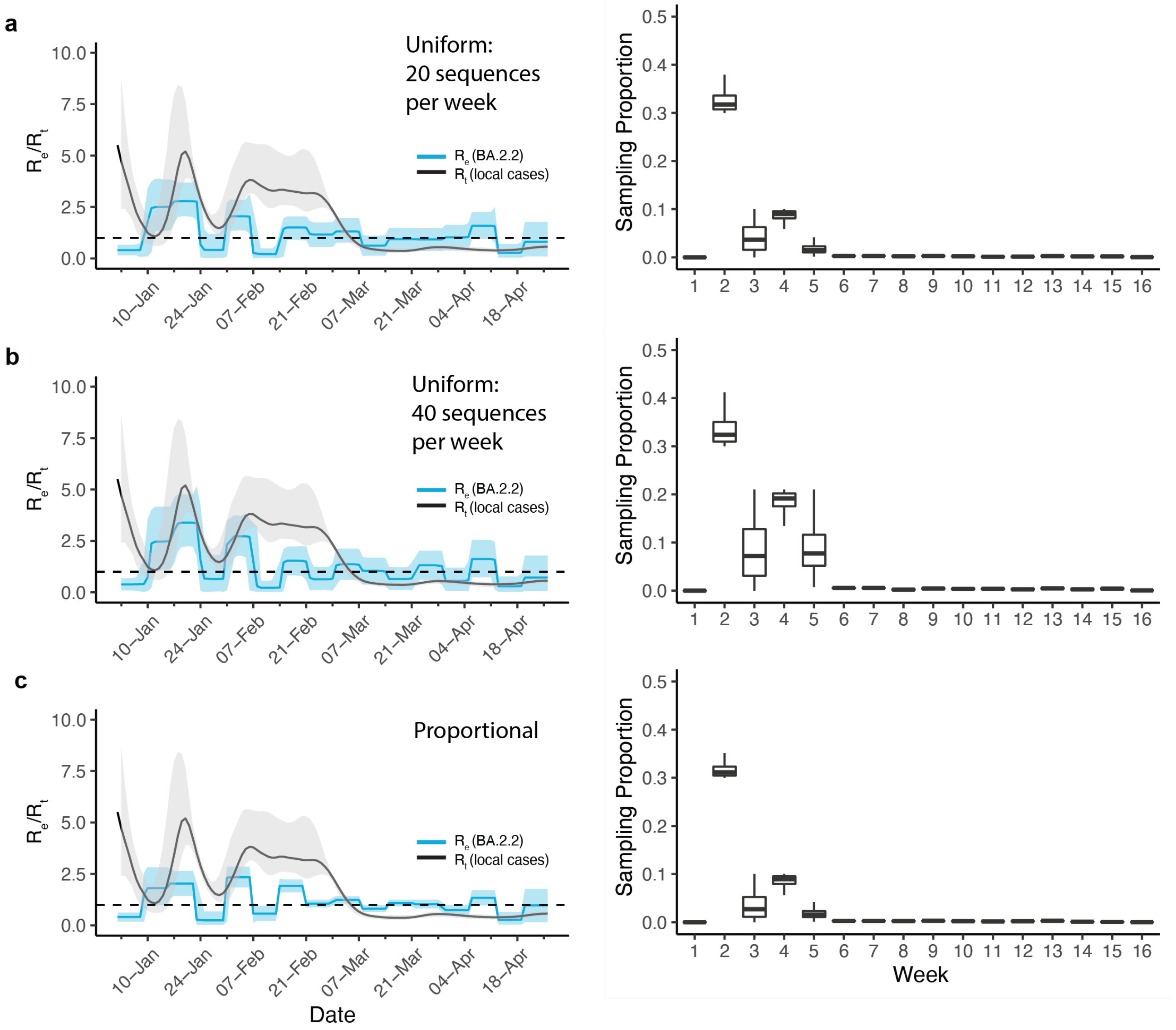


**Supplementary Figure 10** | The effective reproductive number ($R_e$) and the posterior distribution of sampling proportion over time based on uniform and proportional subsampling datasets. (a) Uniform sampling with 20 sequences per week. (b), Uniform sampling with 40 sequences per week. (c) Proportional sampling with 10 sequences per week (2-5 weeks) and 0.2% of the number of incident cases per week after 6th week. The instantaneous effective reproduction number ($R_t$) is shown in black line. The shaded area denotes the 95% confidence interval. In boxplots, the median is indicated by the band, the first and third quartiles by the box, and the whiskers indicate the interquartile range by 1.5.

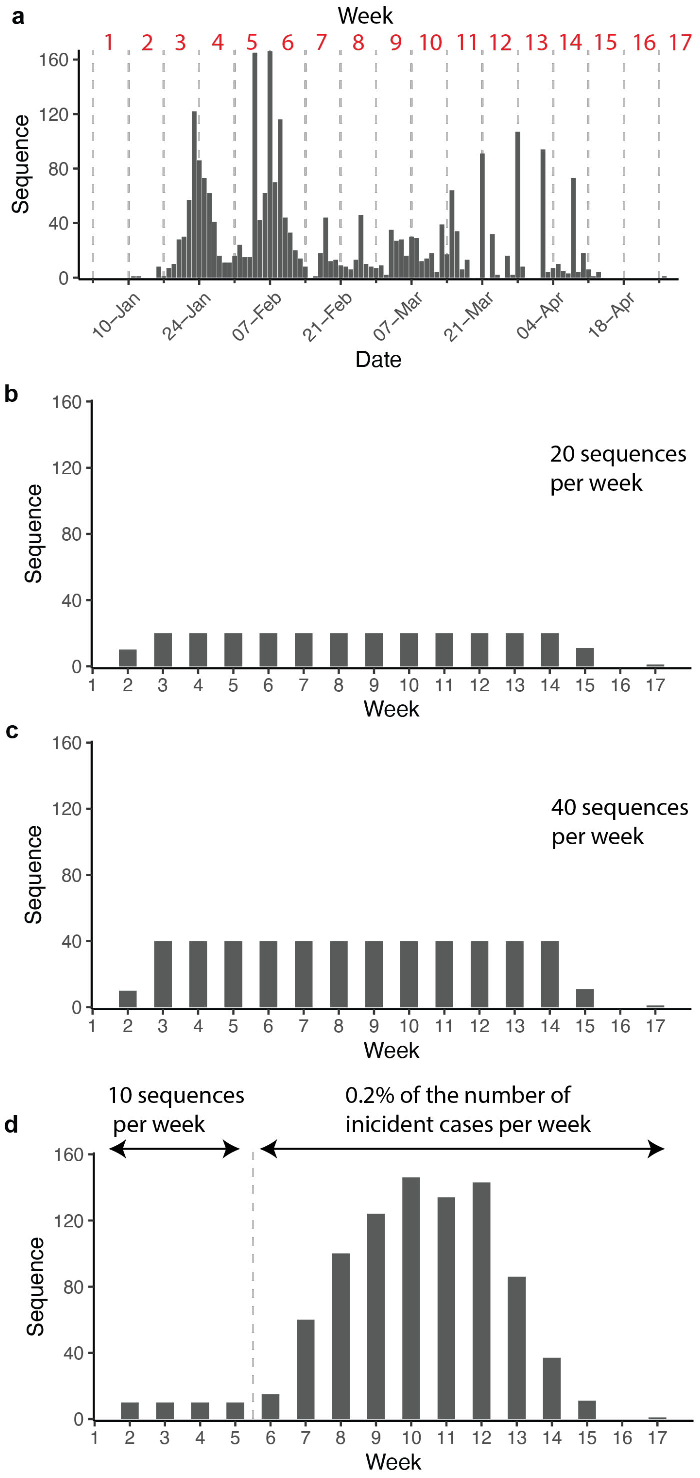


**Supplementary Figure 11** | Summary of HK-BA.2.2 sequences using different sampling schemes. The number of HK-BA.2.2 sequences over time from Jan-April 2022 using no sampling strategy (a), uniform sampling with 20 sequences per week (b), uniform sampling with 40 sequences per week (c) and proportional sampling with 10 sequences per week (2-5 weeks) and 0.2% of the number of incident cases per week after 6th week (d). When the number of available sequences is less than the case value based on the sampling strategy per week, the maximum number of sequences will be used.

## Supplementary Tables:

**Supplementary Table 1** | A summary of the number of local cases and BA.2.2 sequences per week.

| Week | Date | Local cases | All[a] | | Uniform: 20[b] | | Uniform: 40[c] | | Proportional[d] | |
|---|---|---|---|---|---|---|---|---|---|---|
| | | | BA.2.2 sequences | Sampling proportion | BA.2.2 sequences | Sampling proportion | BA.2.2 sequences | Sampling proportion | BA.2.2 sequences | Sampling proportion |
| 2 | 2022-01-10 to 2022-01-16 | 33 | 10 | 30.30% | 10 | 30.30% | 10 | 30.30% | 10 | 30.30% |
| 3 | 2022-01-17 to 2022-01-23 | 200 | 255 | 127.50% | 20 | 10.00% | 40 | 20.00% | 10 | 5.00% |
| 4 | 2022-01-24 to 2022-01-30 | 684 | 300 | 43.86% | 20 | 2.92% | 40 | 5.85% | 10 | 1.46% |
| 5 | 2022-01-31 to 2022-02-06 | 1,229 | 339 | 27.58% | 20 | 1.63% | 40 | 3.25% | 10 | 0.81% |
| 6 | 2022-02-07 to 2022-02-13 | 7,543 | 463 | 6.14% | 20 | 0.27% | 40 | 0.53% | 15 | 0.20% |
| 7 | 2022-02-14 to 2022-02-20 | 29,783 | 96 | 0.32% | 20 | 0.07% | 40 | 0.13% | 60 | 0.20% |
| 8 | 2022-02-21 to 2022-02-27 | 166,712 | 100 | 0.06% | 20 | 0.01% | 40 | 0.02% | 100 | 0.06% |
| 9 | 2022-02-28 to 2022-03-06 | 436,386 | 124 | 0.03% | 20 | 0.00% | 40 | 0.01% | 124 | 0.03% |
| 10 | 2022-03-07 to 2022-03-13 | 235,174 | 146 | 0.06% | 20 | 0.01% | 40 | 0.02% | 146 | 0.06% |
| 11 | 2022-03-14 to 2022-03-20 | 156,378 | 134 | 0.09% | 20 | 0.01% | 40 | 0.03% | 134 | 0.09% |
| 12 | 2022-03-21 to 2022-03-27 | 80,785 | 143 | 0.18% | 20 | 0.02% | 40 | 0.05% | 143 | 0.18% |
| 13 | 2022-03-28 to 2022-04-03 | 42,865 | 213 | 0.50% | 20 | 0.05% | 40 | 0.09% | 86 | 0.20% |
| 14 | 2022-04-04 to 2022-04-10 | 18,725 | 120 | 0.64% | 20 | 0.11% | 40 | 0.21% | 37 | 0.20% |
| 15 | 2022-04-11 to 2022-04-17 | 7,547 | 11 | 0.15% | 11 | 0.15% | 11 | 0.15% | 11 | 0.15% |
| 16 | 2022-04-18 to 2022-04-24 | 3,930 | 0 | 0.00% | 0 | 0.00% | 0 | 0.00% | 0 | 0.00% |
| 17 | 2022-04-25 to 2022-04-31 | 2,244 | 1 | 0.04% | 1 | 0.04% | 1 | 0.04% | 1 | 0.04% |

[a] total number of available BA.2.2 sequences from Hong Kong
[b] subsampled dataset for $R_e$ estimation using a uniform subsampling strategy of 20 sequences per week
[c] subsampled dataset for $R_e$ estimation using a uniform subsampling strategy of 40 sequences per week
[d] subsampled dataset for $R_e$ estimation using a subsampling strategy proportional to reported cases

**Supplementary Table 2** | The ratio of the number of non-synonymous and synonymous substitutions per site ($d_N/d_S$) of the BA.2.2, BA.2.3.1, BA.2.8, BA.2.12.1, BA.4 and BA.5 lineages.

| PANGO Lineage | Dominated Region[a] | $d_N/d_S$ (FEL) | $d_N/d_S$ (SLAC) | Circulation distributions[b] |
|---|---|---|---|---|
| **BA.2.2** (*215*) | Hong Kong | 0.57 | 0.61 | Hong_Kong 78.0%, Australia 11.0%, United Kingdom 5.0%, China 1.0%, United States of America 1.0% |
| **BA.2.3.1** (*57*) | Japan | 0.51 | 0.56 | Japan 100.0%, Canada 0.0%, United States of America 0.0%, Singapore 0.0%, Vietnam 0.0% |
| **BA.2.8** (*160*) | Scotland | 0.41 | 0.45 | United Kingdom 94.0%, United States of America 2.0%, Germany 1.0%, Denmark 1.0%, Israel 0.0% |
| **BA.2.12.1** (*218*)* | USA | 0.75 | 0.78 | United States of America 84.0%, Canada 6.0%, United Kingdom 2.0%, Denmark 1.0%, Israel 1.0% |
| **BA.4** (*188*) | South Africa | 1.42 | 1.37 | United States of America 29.0%, United Kingdom 26.0%, South_Africa 9.0%, Denmark 5.0%, Israel 4.0% |
| **BA.5** (*55*) | South Africa | 1.72 | 1.65 | United States of America 36.0%, United Kingdom 15.0%, Luxembourg 10.0%, Denmark 6.0%, Germany 4.0% |

*Numbers in bracket on first column represents the number of sequences used.*
**Total number of unique spike sequences from this sublineage was 1590, 218 sequences were subsampled from each major clade*
[a]*According to Pango nomenclature (cov-Lineages.org) accessed on 30/04/2022*
[b]*The proportions of sequences for each lineage in different regions (cov-Lineages.org, accessed on 14/7/2022)*

Supplementary Data:

**Dataset 1** | Summary of BA.1.* monophyletic clades in Hong Kong.

**Dataset 2** | Summary of Delta monophyletic clades in Hong Kong.

**Dataset 3** | Summary of BA.2.* monophyletic clades in Hong Kong.

**Dataset 4** | Acknowledgements to sequences obtained from GISAID (assessed on 01-May-2022).

Reference


1. Organisation, W.H., *Guidance For Surveillance of SARS-CoV-2 Variants Interim Guidance. (World health organisation, 2021).*
2. Cori, A., et al., *A new framework and software to estimate time-varying reproduction numbers during epidemics.* Am J Epidemiol, 2013. **178**(9): p. 1505-12.
3. Parag, K.V., *Improved estimation of time-varying reproduction numbers at low case incidence and between epidemic waves.* PLoS Comput Biol, 2021. **17**(9): p. e1009347.
4. Bosse, N., S. Abbott, and F.S. EpiForecasts, *Scoringutils: Utilities for scoring and assessing predictions.* 2020.
5. Jordan, A., F. Krüger, and S. Lerch, *Evaluating Probabilistic Forecasts with scoringRules.* Journal of Statistical Software, 2019. **90**(12): p. 1 - 37.
6. Kosakovsky Pond, S.L. and S.D. Frost, *Not so different after all: a comparison of methods for detecting amino acid sites under selection.* Mol Biol Evol, 2005. **22**(5): p. 1208-22.
7. Viana, R., et al., *Rapid epidemic expansion of the SARS-CoV-2 Omicron variant in southern Africa.* Nature, 2022. **603**(7902): p. 679-686.
