# Supplementary material for: Resurgence of Omicron BA.2 in SARS-CoV-2 infection-naive Hong Kong
Source: Nat Commun. 2023 Apr 27;14:2422. doi: 10.1038/s41467-023-38201-5 (PMC10134727; doi:10.1038/s41467-023-38201-5)
Supplement: Supplementary file 3 — Description of Additional Supplementary Files [file 41467_2023_38201_MOESM3_ESM.pdf]

Description of Additional Supplementary Files

File Name: Supplementary Data 1
Description: Summary of BA.1.* monophyletic clades in Hong Kong.

File Name: Supplementary Data 2
Description: Summary of Delta monophyletic clades in Hong Kong.

File Name: Supplementary Data 3
Description: Summary of BA.2.* monophyletic clades in Hong Kong.

File Name: Supplementary Data 4
Description: Acknowledgements to sequences obtained from GISAID (assessed on 01-May-2022).
