# Supplementary material for: Resurgence of Omicron BA.2 in SARS-CoV-2 infection-naive Hong Kong
Source: Nat Commun. 2023 Apr 27;14:2422. doi: 10.1038/s41467-023-38201-5 (PMC10134727; doi:10.1038/s41467-023-38201-5)
Supplement: Supplementary file 7 — Supplementary Data 4 [file 41467_2023_38201_MOESM7_ESM.pdf]

SUPPLEMENTAL TABLE

Data Availability

GISAID Identifier: EPI_SET_220901sn
doi: 10.55876/gis8.220901sn

All genome sequences and associated metadata in this dataset are published in GISAID's EpiCoV database. To view the contributors of each individual sequence with details such as accession number, Virus name, Collection date, Originating Lab and Submitting Lab and the list of Authors, visit 10.55876/gis8.220901sn

Data Snapshot

- EPI_SET_220901sn is composed of 8,452 individual genome sequences.
- The collection dates range from 2019-12-26 to 2022-05-01;
- Data were collected in 153 countries and territories;
- All sequences in this dataset are compared relative to hCoV-19/Wuhan/WIV04/2019 (WIV04), the official reference sequence employed by GISAID (EPI_ISL_402124). Learn more at https://gisaid.org/WIV04.
